# *Bacillus velezensis* CNPMS-22 as biocontrol agent of pathogenic fungi and plant growth promoter

**DOI:** 10.3389/fmicb.2025.1522136

**Published:** 2025-03-05

**Authors:** José Edson Fontes Figueiredo, Gisele de Fátima Dias Diniz, Mikaely Sousa Marins, Felipe Campos Silva, Vitória Palhares Ribeiro, Fabrício Eustáquio Lanza, Christiane Abreu de Oliveira-Paiva, Valter Cruz-Magalhães

**Affiliations:** ^1^Biochemistry Molecular Laboratory, Embrapa Maize and Sorghum Research Center, Sete Lagoas, Brazil; ^2^Soil Microbiology Laboratory, Embrapa Maize and Sorghum Research Center, Sete Lagoas, Brazil; ^3^Universidade Federal de Viçosa, Viçosa, Brazil; ^4^Laboratory of Phytopathology, Embrapa Maize and Sorghum Research Center, Sete Lagoas, Brazil; ^5^Microbial Ecology Laboratory, Embrapa Maize and Sorghum Research Center, Sete Lagoas, Brazil

**Keywords:** phytopathogenic fungi, biofungicide, maize yield, genome sequencing, bioinoculants

## Abstract

**Introduction:**

*Bacillus velezensis* is a ubiquitous bacterium with potent antifungal activity and a plant growth promoter. This study investigated the potential of *B. velezensis* CNPMS-22 as a biocontrol agent against phytopathogenic fungi under diverse experimental conditions and its potential as a plant growth promoter. Genome sequencing and analysis revealed putative genes involved in these traits.

**Methods:**

This research performed *in vitro* experiments to evaluate the CNPMS-22 antagonistic activity against 10 phytopathogenic fungi using dual culture in plate (DCP) and inverted sealed plate assay (ISP). Greenhouse and field tests evaluated the ability of CNPMS-22 to control *Fusarium verticillioides* in maize plants *in vivo*. The CNPMS-22 genome was sequenced using the Illumina HiSeq 4,000 platform, and genomic analysis also included manual procedures to identify genes of interest accurately.

**Results:**

CNPMS-22 showed antifungal activity *in vitro* against all fungi tested, with notable reductions in mycelial growth in both DCP and ISP experiments. In the ISP, volatile organic compounds (VOCs) produced by CNPMS-22 also altered the mycelium coloration of some fungi. Scanning electron microscopy revealed morphological alterations in the hyphae of *F*. *verticillioides* in contact with CNPMS-22, including twisted, wrinkled, and ruptured hyphae. Eight cluster candidates for synthesizing non-ribosomal lipopeptides and ribosomal genes for extracellular lytic enzymes, biofilm, VOCs, and other secondary metabolites with antifungal activity and plant growth promoters were identified by genomic analysis. The greenhouse and field experiments showed that seed treatment with CNPMS-22 reduced *Fusarium* symptoms in plants and increased maize productivity.

**Conclusion:**

Our findings highlight the CNPMS-22’s potential as bioinoculant for fungal disease control and plant growth with valuable implications for a sustainable crop productivity.

## 1 Introduction

*Bacillus velezensis* is a ubiquitous species commonly isolated from diverse niches (Alenezi et al., 2021). It is harmless to humans and animals, does not pollute the environment, and can be cultured and preserved as colonies or spores (Ye et al., 2018; Alenezi et al., 2021). In the last two decades, several strains of *B. velezensis* have gained popularity as a potential biocontrol agent in agriculture (Koumouts et al., 2004; Vignesh et al., 2022; Hammad et al., 2023; Liu et al., 2024). To date, at least 10 commercial products using *B. velezensis* are available on the market (Ngalimat et al., 2021).

The antimicrobial activity of *B. velezensis* has been well demonstrated by numerous studies *in vitro*, laying a solid foundation for its potential applications. Secondary metabolites produced by *B. velezensis* exhibit broad-spectrum activity against viruses, bacteria, fungi, nematodes, and insects (Rashid et al., 2017; Rabbee and Baek, 2020; Choub et al., 2021; Hu et al., 2022; Diniz et al., 2023; Kamalanathan et al., 2023; Liu et al., 2024). Furthermore, several volatile diffusively organic compounds (VOCs) with potent antimicrobial activity have been identified in many strains of *B. velezensis* (Xu et al., 2016; Lim et al., 2017; Calvo et al., 2020; Li et al., 2020; Ling et al., 2022; Choub et al., 2022; Wu et al., 2023). Consequently, secondary metabolites and VOCs may reduce crop losses with the advantage of being safer and more efficient than chemical fungicides (Gao et al., 2017; Schulz-Bohm et al., 2017; Li et al., 2020; Choub et al., 2022; Ling et al., 2022; Zhao et al., 2022).

Furthermore, the antimicrobial activity of *B. velezensis* is not solely dependent on its lipopeptides, antibiotics, and VOC production. In addition, biofilm, quorum sensing, quorum quenching, proteases, and hydrolytic enzymes play crucial roles in the biocontrol mechanisms (Ling et al., 2022; Tang et al., 2024). In addition to suppressing the growth and development of many phytopathogens, *B. velezensis* produces secondary metabolites as quorum sensing and quorum quench that trigger plant defense mechanisms, thus enhancing their ability to respond to pathogens and pest attacks (Fan et al., 2018; Rabbee et al., 2019; Diniz et al., 2023; Jang et al., 2023; Majdura et al., 2023).

This study aimed to evaluate *B*. *velezensis* CNPMS-22’s antagonistic potential against phytopathogenic fungi through *in vitro*, greenhouse, and field experiments and perform the genome sequencing of CNPMS-22 to identify the genes responsible for these attributes.

## 2 Materials and Methods

### 2.1 Material and experimental conditions

The strain CNPMS-22 was isolated in 2007 in an experiment exploring the frequency of endophytic fungi in maize cultivated in the Brazilian Cerrado soil. The bacterium showed strong antagonist activity against the fungus growing in a plate. After obtaining pure colonies, the isolate was labeled CNPMS-22 and deposited in the Embrapa Collection of Multifunctional and Phytopathogenic microorganisms of maize and sorghum. Initially, the bacterium was identified as *Bacillus subtilis* by 16S rDNA sequencing (GenBank accession number MH358457). Compatibility tests *in vitro* were carried out with CNPMS-22 and other bacterial species isolated from maize fields and commercial products based on bioinoculants available on the Brazilian market. This test is routinely made at the Embrapa microbial laboratory whenever new bacterial isolates are prospected for agronomic uses. Since then, the isolate has been the subject of extensive study to evaluate its potential as an antagonist of pathogenic fungi and a plant growth promoter. *In vitro* experiments tested the CNPMS-22 antagonist activity against 10 pathogenic fungi and the four most virulent races of *F*. *verticillioides* in Brazilian maize crops. The following fungi were used in dual culture in plate (DCP) and volatile organic compounds (VOCs) assays: *Aspergillus niger* F22, *A. ochraceus* EMS301, *Colletotrichum graminicola* EMS132, *Fusarium graminearum* EMS142, *F*. *paranaense* CML4246, *F*. *phaseoli* CML4255, *F*. *verticillioides* CML2778, *Macrophomina phaseolina* EMS297, *Penicillium* sp. EMS300, and *Stenocarpella maydis* EMS299. The Brazilian maize variety BRS Caimbé was used in the greenhouse and field experiments. The experiments were conducted at the Embrapa Maize and Sorghum Research Center in Sete Lagoas (Latitude: −19.4679, Longitude: −44.2477 19° 28′ 4″ South, 44° 14′ 52″ West), state of Minas Gerais, Brazil. The raw data that gave rise to the analysis are presented in Supplementary file S2.

### 2.2 *In vitro* antagonist activity of CNPMS-22 against fungi by dual culture in plate and inverted sealed plate

The dual culture in plate assay (DCP) tested the CNPMS-22 antagonist activity against 10 phytopathogenic fungi in Potato Dextrose Agar (PDA) medium (Beever and Bollard, 1970). Briefly, a 5 mm disc from the edge of pure cultures of each pathogen, grown for 10 days in PDA, was transferred to the center of a Petri dish of 90 mm diameter containing the PDA medium. The CNPMS-22 was grown for 48 h at 28°C with constant agitation at 120 rpm. The inoculum concentration was adjusted to 1 × 10^8^ CFU.ml^−1^ by optical density readings (OD = 1) at a wavelength of 540 nm in a spectrophotometer. Promptly, aliquots of 20 μL of the standardized culture were inoculated in four equidistant points from the center of the plate. The test was carried out in quadruplicate, and the control consisted of pure cultures of the phytopathogens. The colony radius of each fungus was measured when the growth of the control reached the entire surface of the medium.

The inverted sealed plates assay evaluated the effect of VOCs on fungal growth (Gao et al., 2017). A 20 μL of the standardized culture of CNPMS-22 (1 × 10^8^ CFU.ml^−1^) was plated on a PDA medium and incubated at 28°C for 48 h. Subsequently, mycelial discs of pathogens previously grown in PDA were placed in the center of new Petri dishes containing the PDA medium. Then, the lid of each plate was removed and placed face to face. The top plate contained PDA inoculated with tested fungi, and the bottom plate was inoculated with CNPMS-22. The two dishes were taped with parafilm and incubated at 28°C for 10 days. The controls consisted of each tested fungus inoculated in a PDA medium (top plate) and the bottom plate with PDA alone, without CNPMS-22 inoculation.

All plates in both experiments were subjected to constant temperature (28°C), with a 12 h light photoperiod, and humidity, in a bacteriological incubator oven (Solab, Piracicaba, SP, Brazil). The experiment was monitored daily, and during the approximately 10 days of the experiment, both the bacterium and fungi continued to grow in the control plates, indicating that sufficient nutrients were present in the culture media. The plates were photographed, and the diameter of the colonies, color, and the density of mycelium of pathogens were evaluated. The experiment was performed in triplicate.

For both assays, the percentage of inhibition (%), which indicates the area of the fungus’s mycelium growth, was calculated using the formula proposed by Abbott (1925):


$$\mathrm{PI}\;\left(\%\right)=\frac{\left(T-t\right)\;x\;100}{T}$$

Where: |PI| = percent inhibition; T = mycelium radius in the absence of the antagonist; t = mycelium radius in the presence of the antagonist.

### 2.3 Scanning electron microscopy of the antagonistic effect of CNPMS-22 on *Fusarium verticillioides* morphology

A pure culture of CNPMS-22 was mixed with a spore suspension of 1 × 10^6^ spores.ml^−1^ of *F*. *verticillioides* CML2778 and incubated at 200 rpm for 5 days at 28°C. Then, the dual culture was fixed in a Karnovsky solution (Karnovsky, 1965) by mixing in a 1:1 ratio. After, the samples were stored at 4°C for 24 h. The coverslips were prepared by applying 5 μL of poly-L-lysine (0.1%) and adding 20 μL of each sample once the poly-L-lysine had dried. Then, the samples were washed three times in cacodilate buffer for 10 min each wash, followed by dehydration in acetone 25, 50, 75, 90, and 100%, with one wash for 10 min for each concentration up to 90 and three washes in 100% acetone. Next, the samples were dried at a critical point (CPD 030—BalTec Corporation) and metalized in a gold evaporator (BalTec—SCD 050). The observations were done in a Scanning Electron Microscope FEG-SEM (Field Emission Gun—Scanning Electron Microscope) with an ultra-high resolution, field-free CLARA model 2021 (TESCAN, São Bernardo do Campo, SP, Brazil). The experiment was conducted at the Microscopy Laboratory Electronics and Ultrastructural Analysis (LME) at the Federal University of Lavras, Minas Gerais, Brazil. The experiment was performed in triplicate, and 10 SEM images were considered for analysis. The parameters evaluated were wrinkled and broken hyphae and biofilm associated with the hyphae of *F*. *verticillioides*. The data analysis was made by counting the number of events per image and calculating the percentage of the events observed in each treatment (confrontation and control).

### 2.4 Biofilm formation

Two methods were used to test the CNPMS-22 biofilm formation capacity. The first method was performed in Eppendorf tubes, according to Latorre et al. (2016), and the second test was performed using the crystal violet staining method described by O’Toole and Kolter (1998).

First, a colony of CNPMS-22 was inoculated in LB medium and grown overnight at 30°C; the concentration was adjusted to 1 × 10^9^ CFU.ml^−1^ after spectrophotometric reading (OD 595). After that, a 1/20 dilution was transferred to Eppendorf tubes of 1.5 mL containing 600 μL of nutrient broth medium or polypropylene tubes of 10 mL containing 2.5 mL of the culture medium. After 24 h of incubation at 30°C without shaking, the medium was gently discarded, and the tubes were washed three times with deionized water (Latorre et al., 2016). Following, 1 mL of 1% crystal violet solution was added to each tube and incubated for 45 min at room temperature. After the dye was discarded, the tubes were gently washed three times, and the dye adhered to the biofilm solubilized with 1 mL absolute ethanol, and read in a spectrophotometer (OD 600). The color intensity and size of the adherent cells via crystal violet staining were used as parameters for the qualitative measurement of biofilm synthesis. The color intensity and size of the adherent crystal violet ring were scored from faint (−) to strong (++), as described by Fall et al. (2004). The experiment was performed in quintuplicates and repeated three times.

The second method to evaluate the biofilm formation capacity by CNPMS-22 was based on Stepanović et al. (2007) with modifications. In triplicate, 10 μL of the standardized bacterial culture at 1 × 10^8^ CFU.ml^−1^ were inoculated in 200 μL of TSB culture medium with 1% glucose in a polystyrene microplate. For fungi, 20 μL of the culture grown in a TSB liquid medium was inoculated. Then, four 8 mm discs of fungal mycelium were plated in 50 mL of TSB and grown for 12 days at a temperature of 30°C. The negative control consisted of only the culture medium. The microplate was incubated for 48 h at 30°C. After this period, the culture was removed by inversion, the wells were washed three times with 200 μL of distilled water, and the plate was placed inverted on a paper towel for complete drainage. An aliquot of 200 μL methanol was added to each well to fix the biofilm. After 20 min, the methanol was removed, and the plate was left at room temperature until drying. Then, 200 μL of 0.5% crystal violet solution was added to each well, and after 15 min, the dye was removed by inversion. The plate was washed with running distilled water and placed at room temperature to dry. Finally, 200 μL of ethyl alcohol was added to each microplate well and rested for 30 min. The microplate was read in a UV/VIS spectrophotometer (FLUOstar Omega, BMG LABTECH, Germany) at 570 nm.

The classification criterion for determining the biofilm formation capacity was based on the following optical density values: as non-forming (Doa ≤ Docn), weakly forming (Docn ≤ Doa ≤ 2x Docn), moderately formative (2x Docn < Doa ≤ 4x Docn), or strong formative (4x Docn < Doa). Where: Doa = Optical density of the absorbance and Docn = Optical density of the negative control.

### 2.5 Exopolysaccharides production

The evaluation of exopolysaccharides (EPS) production by CNPMS-22 was carried out according to the methodology of Paulo et al. (2012). Briefely, the culture medium was composed of 50 g.l^−1^ sucrose, 20 g.l^−1^ yeast extract, 20 g.l^−1^ MgSO_4_, 0.01 g.l^−1^ NaCl, 0.01 g.l^−1^ FeSO_4_, 0.01 g.l^−1^ MnSO_4_, 0.02 g.l^−1^ of CaCl_2_ and 20 g.l^−1^ of K_2_HPO_4_, pH 7.5 (Guimarães et al., 1999). Filter paper discs (Whatman 42) with 5 mm diameter were sterilized and applied to the culture medium. Then, 5 μL of the bacterial culture grown in TSB medium was standardized to 1 × 10^8^ CFU.ml^−1^ and inoculated on the plates. The plates were incubated at 30°C for 24 h, and we assessed EPS production based on the absence or presence of mucous colonies around the discs.

A translucent or creamy material surrounding the colonies indicates the potential EPS production. EPS production was confirmed by scraping the culture using a platinum loop and mixing it with 2 mL of absolute ethanol. The formation of precipitate was considered a positive result, and the presence of turbidity was considered a negative result (Paulo et al., 2012).

### 2.6 Antifungal activity of CNPMS-22 against *Fusarium verticillioides* in the greenhouse

Spores of a pure culture of *F*. *verticillioides* CML2778 were collected and transferred to a PDA medium. Erlenmeyer flasks of 1 L capacity containing 200 g of crushed maize grains and 20% humidity were autoclaved twice at 120°C for 30 min. After, discs of CML2778 grown in PDA were inoculated in five Erlenmeyer (two discs per flask) and incubated at 25°C for 20 days in a Biochemical Oxygen Demand (BOD).

Before planting, 100 g of crushed grains containing the fungal inoculum were placed in a 5-liter pot containing red ravine soil (C horizon) and incorporated into the first 5 cm of the soil. The experiment was arranged in a completely randomized design, with six treatments and eight replicates in 48 plastic pots, each with a 5 L capacity and four plants per pot. The treatment consisted of 1- soil infected with CML2778 + seeds treated with CNPMS-22; 2- soil infected with CML2778 + seeds treated with Captan; 3- soil infected with CML2778 + untreated seeds; 4- uninfected soil + seeds treated with the fungicide Captan; 5- uninfected soil + seeds treated with CNPMS-22; 6- uninfected soil with untreated seeds (control). The experiment was irrigated daily, and the temperature in the greenhouse was 28°C. After 30 days, the plants were cut at the base (5 cm from the soil surface) for evaluation. The parameters evaluated were the number of living plants per pot (stand), the height of each plant (cm), measured from the ground to the tip of the last fully open leaf, and the dry mass of the aerial part of each plant (g). The aerial part the plants were weighed, placed in paper bags, dried in an oven at 60°C for 48 h, and weighed again. The data were subjected to analysis of variance (ANOVA) and the Tukey mean comparison test at 5% probability using the SISVAR 5.6 statistical program.

### 2.7 Field experiment

For the field experiments, seeds of maize variety BRS Caimbé, susceptible to *F*. *verticillioides*, were disinfested and inoculated with a mixture of suspensions of 1 × 10^8^ CFU.ml^−1^ of CNPMS-22 and 1 × 10^6^ conidia.ml^−1^ of *F*. *verticillioides* CML2778, both at 0.8% NaCl (w/v). After a two-hour incubation in the CNPMS-22 and phytopathogen suspensions, the excess inoculum was removed, and the seeds were mixed with adhesive and dried in a laminar flow chamber. Control seeds were placed in suspensions containing only conidia of CML2778 or 0.8% NaCl (w/w) saline solution. Additionally, the seeds were chemically treated with the fungicide Fludioxonil + Metalaxyl-M was added to the seeds at a concentration of 1,000 ppm.

The experiments were conducted at Embrapa Maize and Sorghum Research Center in Sete Lagoas, Minas Gerais, Brazil, during the 2022/2023 harvest in typical dystrophic Red Oxisol soil. Planting fertilizations were carried out using 400 kg ha^−1^ of NPK 8–28-16 and top-dressing fertilizations using 200 kg ha^−1^ of urea at 23 and 34 days after planting. The experimental design was completely randomized with three replicates. The plots comprised four rows of 5 meters spaced 0.7 meters apart.

At the end of the cycle, approximately 180 days after planting, the ears were harvested from the entire usable area of the plots, and grain weight and moisture were used to calculate productivity. The data obtained were subjected to analysis of variance (ANOVA). The treatment means were compared using the Scott Knott test at 5% significance (*p* < 0.05).

### 2.8 Genome sequencing of CNPMS-22

Genomic DNA extraction of *Bacillus velezensis* CNPMS-22 was performed by the Wizard Genomic DNA Purification Kit (Promega, United States) and quantified on the Qubit^®^ 2.0 fluorometer (Life Technologies).

The library quality was checked in GelBot (Loccus) (Cotia, SP, Brazil), and the genome was sequenced using the Illumina NextSeq1000 platform Illumina with a P1-600 kit in a paired-end strategy by GoGenetic Company (Curitiba, PR, Brazil). The Trimmomatic v. Zero.39 software was used to check the quality of the sequences generated (Bolger et al., 2014). The genome was assembled using the raw reads by SPAdes v. 3.12.0 (Bankevich et al., 2012). The assembly quality was checked by Quast v.5.0.2 (Mikheenko et al., 2018), and the completeness was evaluated using BUSCO (Benchmarking Universal Single-Copy Orthologs v.5.3.1) (Manni et al., 2021). The genome annotation was performed with the Prokka tool v.1.14.6 (Seemann, 2014), the NCBI Prokaryotic Genome Annotation Pipeline (PGAP) v6.6 (Tatusova et al., 2016), and manual annotations.

The pre-assembled genomic sequences were annotated with PROKKA version 1.8 (Seemann, 2014) and RAST version 2.0 (Rapid Annotation using Subsystem Technology) software (Aziz et al., 2008). We also manually analyzed by searching for identities between CNPMS-22 nucleotide sequences and DNA sequences of *B. velezensis*, *B. amyloliquefaciens*, and other bacterial species deposited in the GenBank. The assembled genome of CNPMS-22 was deposited in the GenBank database with the following accession number: JBHGBV000000000.1.

## 3 Results

### 3.1 *In vitro* antagonist test by dual culture in plate and inverted sealed plate

**Figure 1:**
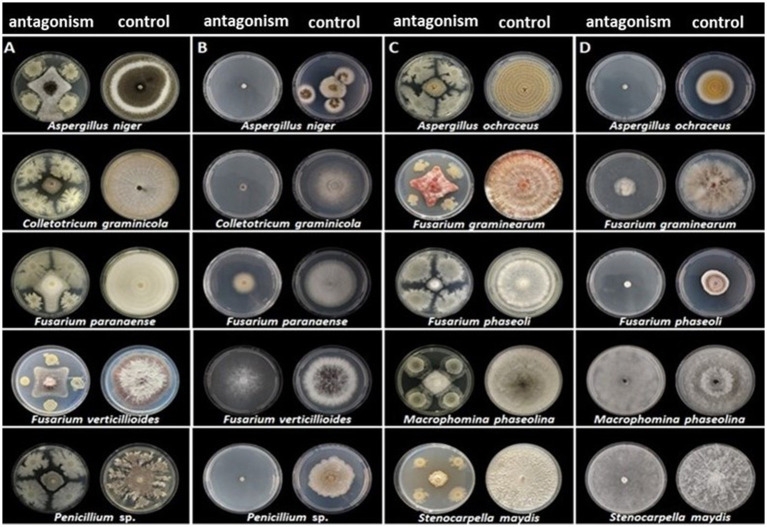
Antagonistic activity of CNPMS-22 against phytopathogenic fungi. **(A,C)** Confrontation by dual culture in plate assay **(B,D)** and organic volatile compounds (VOCs) activity by inverted plate assay.

**Figure 2:**
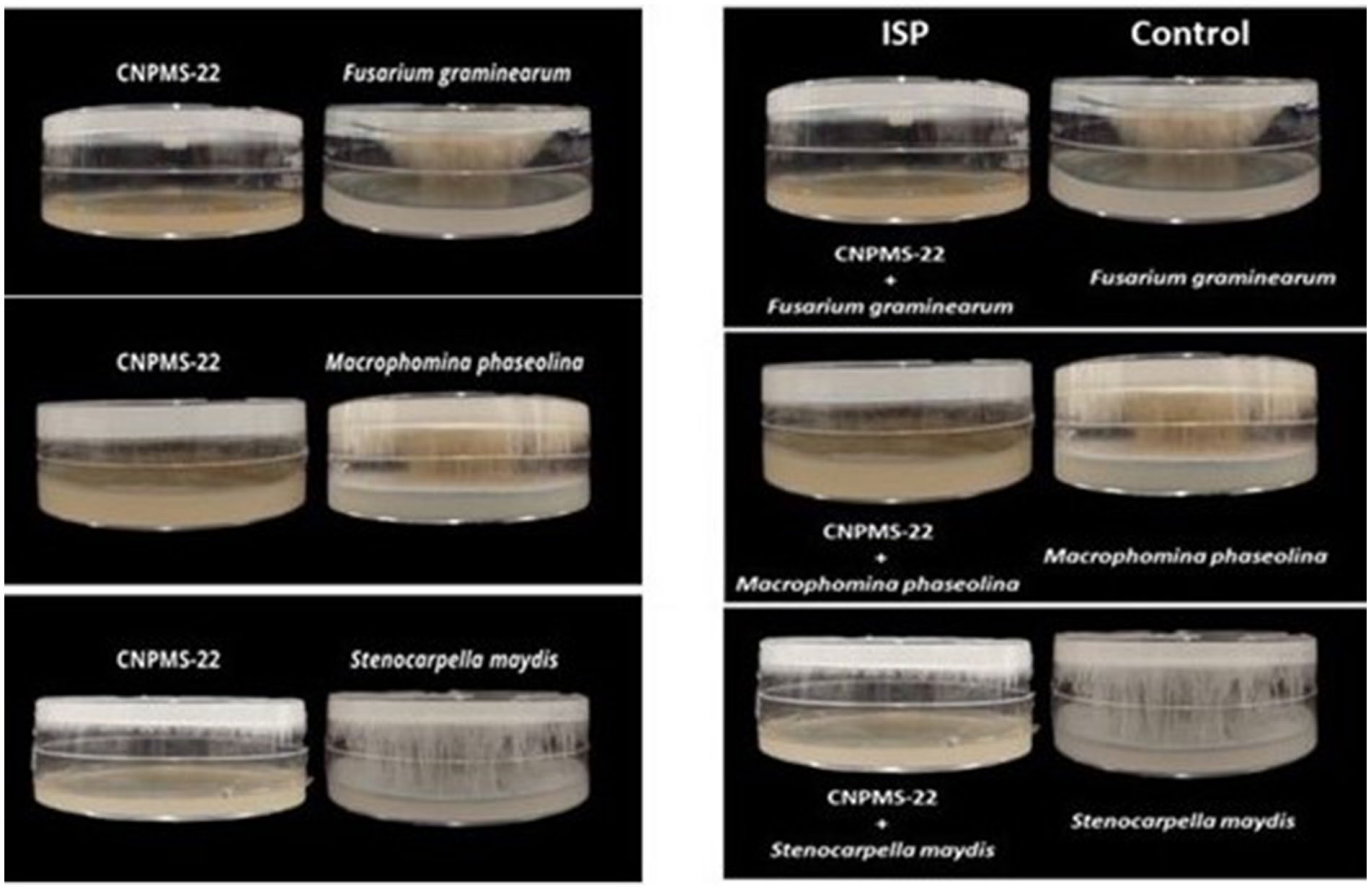
Side view of plates showing the antifungal effect of volatile organic compounds (VOCs) of CNPMS-22. The bacterium reduced the mycelial growth of three species of fungi that grew across the plate’s entire diameter during the experiment’s duration (10 days).

The strain CNPMS-22 exhibited antagonistic activity against the 10 phytopathogenic fungi tested (Figure 1; Table 1). The DCP treatment led to a remarkable reduction in the mycelium growth of all 10 fungi tested, with inhibition rates ranging from 54.50% (*F*. *verticillioides* CML2778) to 76.19% (*Stenocarpella maydis* EMS299). The VOCs produced by CNPMS-22 reduced the radial growth and biomass of all fungi tested, with inhibition rates ranging from 0% (*Macrophomina phaseolina* and *S. maydis*) to 97.60% (*Aspergillus ochraceus*) (Figure 1; Table 1). However, although the mycelium radial growth of *M. phaseolina* EMS297 and *S*. *maydis* EMS299 reached the edges of the plates (Table 1), the mycelial biomass of both fungi was drastically reduced (Figure 2). The VOCs produced by CNPMS-22 also affected the color of the mycelium pigmentation of six of the 10 fungi tested, resulting in specific color changes (Table 1).

**Table 1:** Antagonistic effect of CNPMS-22 on fungal morphology and growth by dual culture in plate and volatile organic compounds assays.

	Dual culture in plate (DCP)		Inverted sealed plates (ISP)			
			(volatile organic compounds)			
Genera/Species	Colony radius	ZI%*	Mycelial growth (cm)	Inhibition (%)	Mycelial density	Hyphae color
*Aspergillus niger* F22	4.20		2.53		+++++	White
F22 + CNPMS22	1.70	59.05	0.16	93.68	−	White
*A. ochraceus* EMS301	3.80		4.16		+++++	Beige
EMS301 + CNPMS22	1.10	69.74	0.10	97.60	−	Beige
*Fusarium graminearum* EMS142	4.20		6.80		+++++	Orange
EMS142 + CNPMS22	1.60	60.71	2.80	58.82	+	White
*Colletotrichum graminicola* EMS132	3.50		6.43		+++++	White/gray
EMS132 + CNPMS22	0.90	72.83	0.30	95.33	+	White
*F. paranaense* CML4246	4.20		6.76		+++++	Yellow
CML4246 + CNPMS22	1.40	65.00	3.73	44.82	+	Pink
*F. phaseoli* CML4255	3.50		3.66		+++++	Brown
CML4255 + CNPMS22	0.80	75.14	0.56	84.70	+++	White
*F. verticillioides* CML2778	4.00		6.16		+++++	Purple
CML2778 + CNPMS22	1.80	54.50	2.96	51.95	+	White
*Macrophomina phaseolina* EMS297	4.20		8.00		+++++	gray
EMS297 + CNPMS22	1.60	60.24	8.00	0.00	+	White
*Penicillium* sp. EMS300	3.40		4.00		+++++	White
EMS300 + CNPMS22	0.90	71.64	0.13	96.75	−	White
*Stenocarpella maydis* EMS299	4.20	76.19	8.00		+++++	White
EMS299 + CNPMS22	1.00		8.00	0.00	+	White

*Zone of Inhibition; mycelial density: +++++ = 100%; ++++ = <80%; +++ = <60%; ++ = < 40%; + = < 20%.

### 3.2 Antagonist effect of CNPMS-22 on *Fusarium verticillioides* morphology by scanning electron microscopy

**Figure 3:**
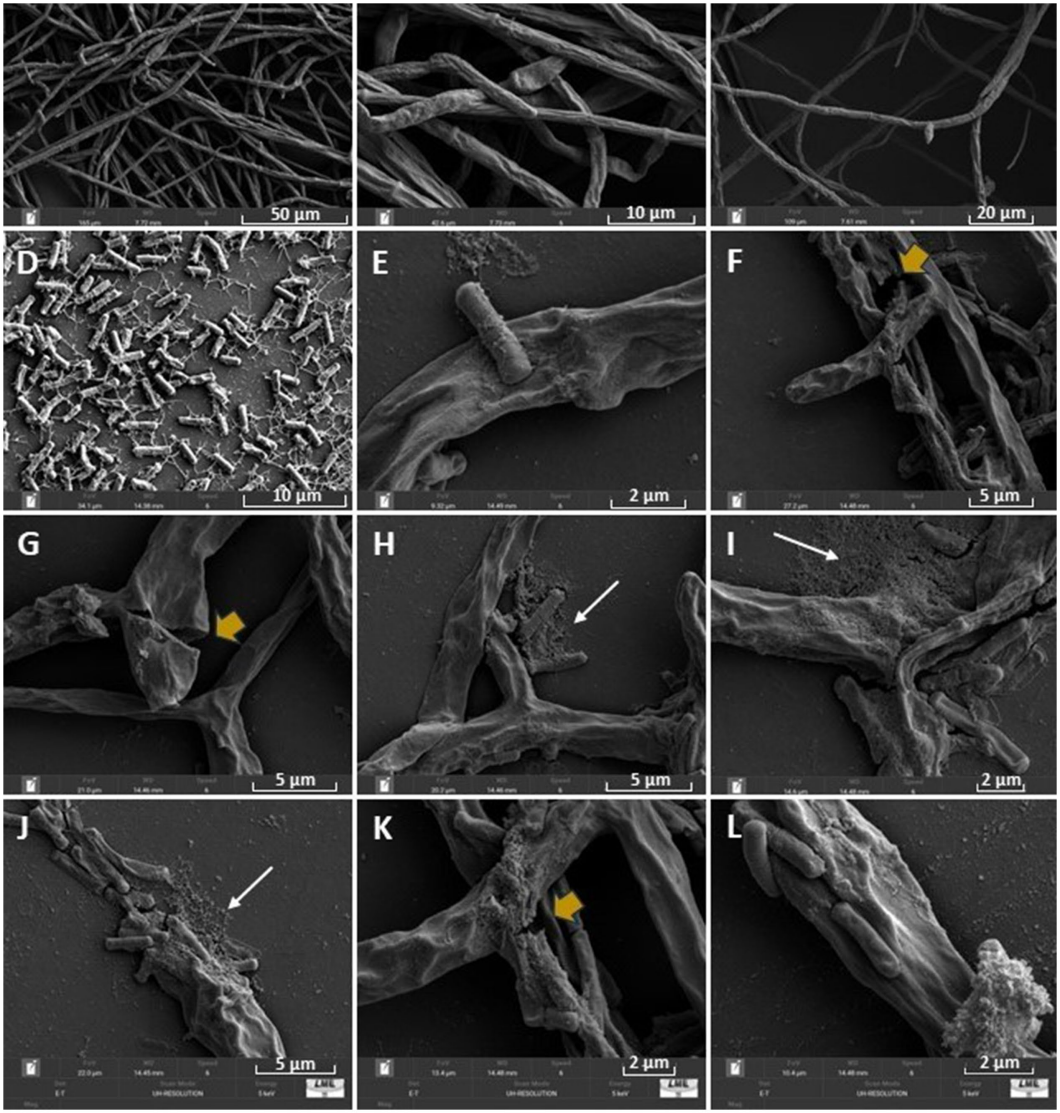
Scanning electron microscopy (SEM) images of CNPMS-22 antagonism against *Fusarium verticillioides*. **(A–C)** Control with an average growth; **(D)** CNPMS-22 showing the typical mesh of extracellular polymeric substances (EPSs) during biofilm formation; **(E,J,L)** CNPMS-22 adhered to wrinkled hyphae of *F*. *verticillioides*, and biofilm; **(F,G,K)** broken and wrinkled hyphae (yellow arrows). (H-J) CNPMS-22 biofilm (white arrows) associated to wrinkled hyphae of *F*. *verticillioides*.

Scanning electron microscopy (SEM) images showed significant morphological alterations in the hyphae of *F*. *verticillioides* CML2778 (Figure 3). The fungus showed twisted, wrinkled, and ruptured hyphae (Figures 3B,C). The biofilm formation by CNPMS-22 was observed by the association between bacterial adhered to the fungus hyphae (Figure 3A). In the treatment of CNPMS-22 + fungus, all hyphae were wrinkled, and the frequency of broken hyphae was 56%. In the control, the frequency of wrinkled hyphae was 2%, and the frequency of broken hyphae was equal to zero.

### 3.3 Biofilm formation

**Figure 4:**
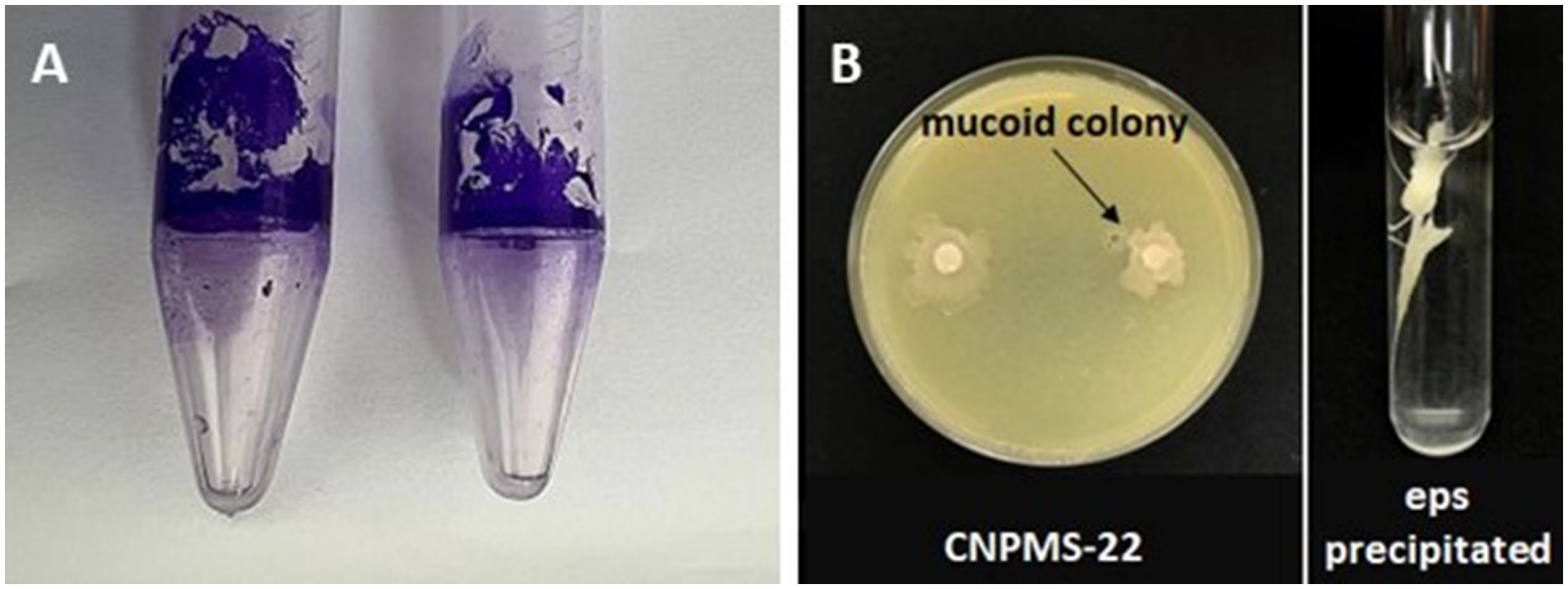
Test of biofilm formation in Eppendorf and EPS production by CNPMS-22. **(A)** Crystal violet ring adhered to the tube, indicating biofilm formation, and **(B)** mucoid colony and extracellular polymeric substances precipitated by absolute ethanol.

The two methods used to demonstrate the biofilm formation by CNPMS-22 were effective (Figure 4). Figure 4A shows the results of the first method described by Latorre et al. (2016). The color intensity and size of the adherent crystal violet ring were scored from faint (−) to strong (++), as described by Fall et al. (2004). The second method based on the crystal violet staining method reported by O’Toole and Kolter (1998) to demonstrate the biofilm ability of a bacterial strain. In Figure 4A, the violet halo around the Eppendorf correspond to a stained biofilm. The results of the second method based on Stepanović et al. (2007), the biofilm formation by CNPMS-22 was classified as very strong (0.30 < Doa) with the value of 3.18 Doa, according to the criteria established by Stepanović et al. (2007).

### 3.4 Exopolysaccharides production

The production of extracellular polymeric substances (EPS) by CNPMS-22 was demonstrated by two tests: the formation of mucoid colony in a plate and precipitation of EPS in absolute ethanol (Figure 4B). The mucoid phenotype in a plate indicated the presence of a thick polysaccharide layer with a matte surface toward the center of the colony and a cream translucent to the edges (Figure 4A).

### 3.5 Greenhouse experiment

**Figure 5:**
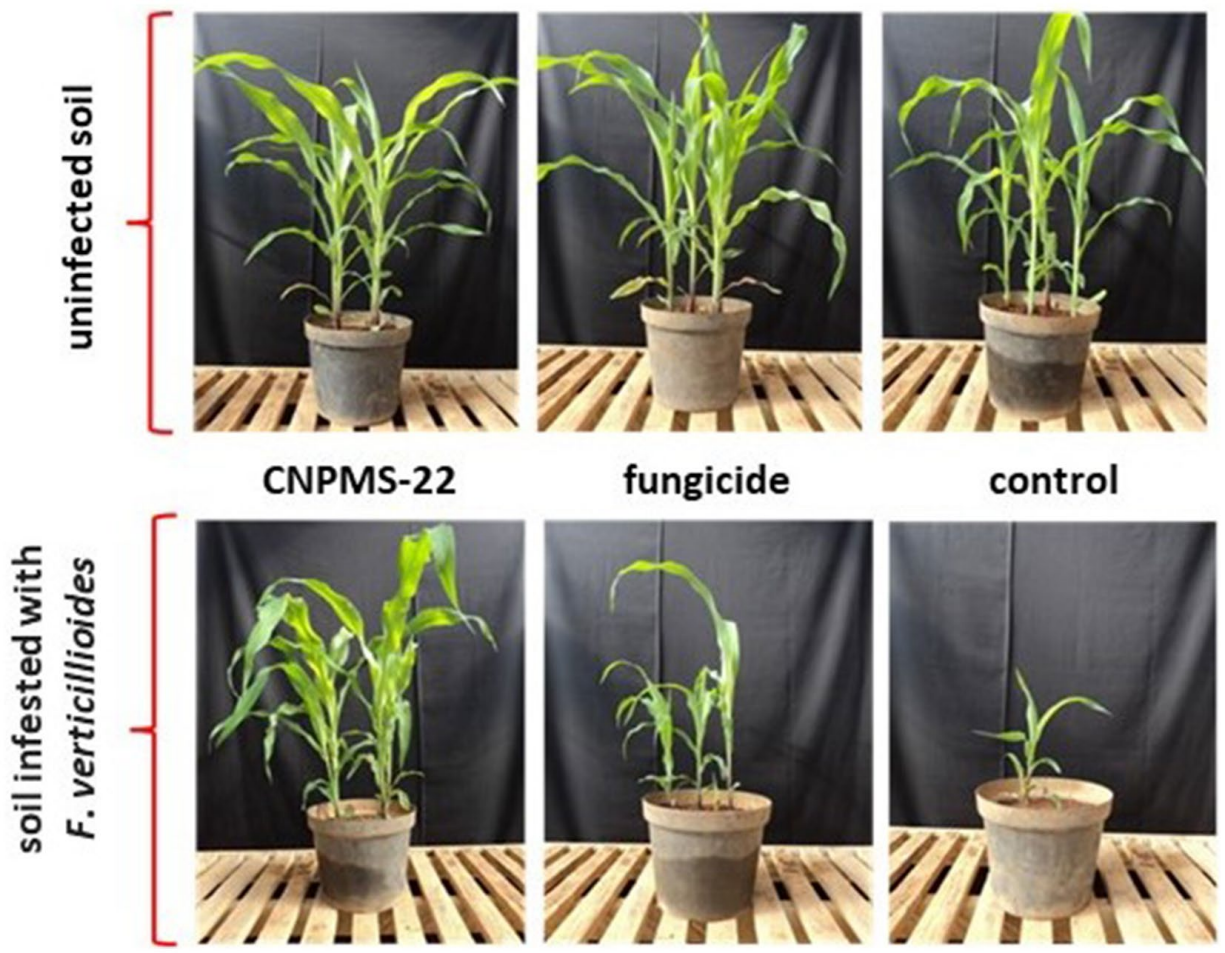
Effect of *Bacillus velezensis* CNPMS-22 and *Fusarium verticillioides* on maize plants growing in the greenhouse.

In the greenhouse, CNPMS-22 inhibits the disease symptoms of *F*. *verticillioides* in 100%, while the plant controls showed apparent disease symptoms (Figure 5; Table 2). The results showed that plants originating from seeds that did not receive treatment showed a reduction in stand height, plant height, and dry weight in the soil infested with *F*. *verticillioides* CML2778. In contrast, seed treatment with CNPMS-22 prevented the reduction of plant stand and dry weight in soil infested with CML2778. In addition, plants from seeds treated with CNPMS-22 showed increased plant height compared to soil without CML2778 infestation. In addition, seed treatment with chemical fungicide was insufficient to prevent the reduction in height and dry weight of plants in soil infested with phytopathogen. The results also showed that seeds treated with CNPMS-22 planted in soil infested with CML2778 promoted an increase in plant height by 32% compared with seeds treated with fungicide (19%), and in plant dry weight by 52%, compared with untreated seeds or treated with fungicide (39%). The experiment carried out in a greenhouse showed that plants originating from seeds without antifungal treatment, germinated in soil artificially infested with CML2778, presented significant mortality. In contrast, seed treatments with the fungicide Captan or CNPMS-22 efficiently controlled seedling death (Table 2). In non-infested soil and soil infested with CML2778, there was no statistical difference in the number of surviving plants at 30 days after planting (DAP) between the treatments with the fungicide Captan or CNPMS-22 (*p* < 0.5) (Table 2).

**Table 2:** Greenhouse experiment to test the antifungal activity of CNPMS-22.

Seed treatment	Stand*		Plant height (cm)		Dry aerial weight (g)	
	Infested soil	Uninfested soil	Infested soil	Uninfested soil	Infested soil	Uninfested soil
Untreated	2.62 Aa	4.00 Ab	65.14 Aa	85.28 Ab	2.48 Aa	3.59 ABb
Fungicide	3.5 Ba	3.37 Aa	72.21 Aa	95.42 Bb	2.71 ABa	4.55 Bb
CNPMS-22	3.12 ABa	4.00 Aa	86.25 Bb	77.91 Aa	3.77 Ba	3.33 Aa

*The average difference in the stand (survival of plants per pot), plant height (measured from the ground to the tip of the last fully open leaf), and dry aerial weight between the different maize seed treatments in infested soil (SI) and soil not infested (SNI) with *Fusarium verticillioides*. Means followed by the same letters did not differ significantly by the Tukey test (*p* < 0.05).

In non-infested soil without any treatment (fungicide or CNPMS-22), all plants survived, while in the same soil that received seeds treated with fungicide or CNPMS-22, plant survival was 85 and 96.75%, respectively. In contrast, in soil infested with CML2778, the plant survival was only 34.37%, while with the application of the fungicide or CNPMS-22, survival rates increased to 87.5% in both treatments (*p* > 0.5).

Plants generated from untreated seeds in infested soil have a statistically lower average height than plants in non-infested soil (control). Concerning seeds treated with the fungicide Captan, it was found that soil infestation negatively influenced the average height of the plants. In contrast, the treatment of seeds with CNPMS-22 positively affected the plants’ height when they were in soil infested, which is statistically different from the others (Table 2).

The analysis of the dry weight of the aerial part of the plants at 30 DAP indicated that the soil infested with CML2778 negatively influenced the development of plants originating from untreated seeds or those treated with fungicide. In contrast, plants from the treatment of seeds inoculated with CNPMS-22 did not show statistically significant differences compared to soil infested with the fungus and non-infested soil (Figure 5; Table 2).

### 3.6 Field experiment

In the field experiment, seeds inoculated with CNPMS-22 efficiently prevented productivity losses due to *F*. *verticillioides* (Table 3). The treatments inoculated with CNPMS-22 and CML2778 showed productivity of 8,340.93 kg ha^−1^, which was statistically equal to the treatment with the chemical fungicide Fludioxonil + Metalaxyl-M (8,219.25 kg ha^−1^) and significantly higher than the controls uninoculated or inoculated with the phytopathogen (7,447.31 and 7,304.60 kg ha^−1^, respectively).

**Table 3:** Productivity of maize grains (kg ha^−1^) as a function of inoculation with CNPMS-22 and *Fusarium verticillioides* after 180 days of field cultivation.

Treatments	Productivity (kg ha^−1^)
Control	7,447.31 b
Control + Fv	7,304.60 b
Fungicide + Fv	8,219.25 a
CNPMS-22 + Fv	8,340.93 a

Fv, *Fusarium verticillioides*; Control, Uninoculated seeds. Means followed by the same letter do not differ by the Scott–Knott test at 5% probability (*n* = 4).

It is essential to highlight that the experiment was done only in one season and locally since our primary objective was to certify that the antagonist activity of CNPMS-22, observed *in vitro*, could be replicated *in vivo* in the greenhouse and field conditions. This result established the foundation for future experiments to transform this strain into a bio-input for tropical maize crops. Embrapa has partnerships with private companies that will assume the costs of future experiments and design and implement new field experiments in the Brazilian maize-producing areas, considering at least two harvest seasons. Only after that can the bio input be commercialized.

### 3.7 Genome sequencing

The genome sequencing of the strain CNPMS-22, previously identified as *Bacillus subtilis*, by the 16S rDNA sequencing (GenBank accession number MH358457), showed high Average Nucleotide Identity (ANI > 95%) with *B. velezensis* (98.772%) and *B. amyloliquefaciens* (98.769%), which represents a difference of only 0.003% between the two genomes. Digital DNA–DNA hybridization and DNA blast at the GenBank dataset showed that CNPMS-22 belongs to *B. velezensis* (100% identity). To confirm the taxonomic position of CNPMS-22, we used the fastANI program (Jain et al., 2018). According to fastANI, the CNPMS-22 strain shares 98.77% nucleotide identity with *B. velezensis*. The digital DNA–DNA hybridization (dDDH) with the Type Strain Genome Server (TYGS) (Meier-Kolthoff and Göker, 2019) and the taxonomic index showed that CNPMS-22 is phylogenetically related to *B. velezensis* FZB42 (former *B. amyloliquefaciens* subsp. plantarum FZB42) (96.4% identity) (NCBI Reference Sequence: NC_009725.2) (Fritze, 2004).

The CNPMS-22 genome was estimated at 4.08 Mbp size, with 4,038 coding sequences for 3,813 proteins, 123 contigs, (>500 pb), 3,096,991 reads, total of bases used 708,45 Mb, medium coverage of 175,982, SSU lengths (1,073 bp-1,538 bp), 84 tRNAs, 14 rRNAs, five ncRNAs, one tmRNA, and 66.49% GC. The assembled genome of CNPMS-22 has been deposited in the GenBank database with the following accession number JBHGBV000000000.1, and the raw data was deposited in the National Library of Medicine under Sequence Read Archive (SRA) SRR31156977, and the extended genomic annotations made by manual analysis are shown in Supplementary Table S1. The genome mining prediction of CNPMS-22 and manual sequence comparisons with nucleotides of bacterial species deposited in the GenBank identified three gene clusters controlling the synthesis of nonribosomal lipopeptides (iturin, surfactin, and fengycin), previously identified by UPLC-MS (Souza et al., 2018). Other gene clusters coding for ribosomal and nonribosomal compounds like proteases, hydrolytic enzymes, and metabolites directly involved in antimicrobial activity were also identified in the CNPMS-22 genome. In addition, genes involved in synthesizing VOCs, biofilm and siderophores, and ISR in plants were also identified in the CNPMS-22 genome (Supplementary Table S1).

## 4 Discussion

### 4.1 DCP and ISP

This study evaluated the *in vitro* antagonist activity of *Bacillus velezensis* strain CNPMS-22 against 10 species of phytopathogenic fungi by dual culture in plate (DCP) and inverted sealed plate (ISP). We also tested the efficacy of the CNPMS-22 for controlling *F*. *verticillioides* in the greenhouse and field experiments. The experiments to evaluate the effect of secreted compounds and VOCs produced by CNPMS-22 showed a significant reduction of the mycelium growth and densities of all fungi tested. Our data endorsed previous studies using *B. velezensis*, which demonstrated the antifungal activity of different strains against various plant-pathogenic fungi (Peypoux et al., 1981; Petrova et al., 2023; Diniz et al., 2024). CNPMS-22 also showed high antagonistic activity against Brazil’s five virulent isolates of *F*. *verticillioides*, *Exserohilum turcicum*, *Acremonium strictum*, *Colletotrichum sublineolum*, and *Stenocarpella maydis* (*Diploidia maydis*) isolated from maize and sorghum cultivated in Brazil (Figueiredo et al., 2010). The results of our study, evaluating the antifungal of CNPMS-22, showed similar inhibition rates with other studies, although the fungi species and experimental conditions were different.

The promising results from the DCP and ISP in this study underscore the potential of CNPMS-22 as a potent biological control agent against various pathogenic fungi of relevant crops. These results show that CNPMS-22 can produce a range of nonribosomal, ribosomal, and volatile organic compounds with antagonistic activity against different fungi.

The VOCs produced by CNPMS-22 also affected the color of the mycelium of six out of ten fungi (*F. verticillioides*, *F*. *graminearum*, *F. paranaense*, *F. phaseoli*, *Colletotrichum graminicola*, and *Macrophomina phaseolina*). Filamentous fungi, particularly ascomycetous, produce secondary metabolites containing a wide variety of pigments, including, e.g., carotenoids, riboflavin, melanins, polyketides, and azaphilones. These pigments determine the characteristic color of each fungus. In *Fusarium* species, the color of mycelium is due to pigment accumulation of the golden yellow polyketide aurofusarin, red rubrofusarin, and the carotenoid neurosporaxanthin, which possibly plays a significant role in the fungus’s yellow or orange color (Cambaza, 2018). *Colletotrichum graminicola* produces melanin synthesized by the pentaketide pathway (Ramachandra et al., 2023). It was observed that white mutants were less efficient than the wild type in their ability to cause plant lesions since melanized appressoria of *C. graminicola* is necessary for fungus penetration in the host plant (Rasmussen and Hanau, 1989). *M. phaseolina* produces melanin that diffuses through hyphae and sclerotia; pigmented *M. phaseolina* is more pathogenic than non-pigmented (Abdallah et al., 2017). These differences have been attributed to melanin, which reduces the susceptibility of melanized microbes to the host defense mechanism (Nosanchuk and Casadevall, 2003). The white-colored hyphae of *C. graminicola* (Rasmussen and Hanau, 1989) and *M. phaseolina* (Abdallah et al., 2017; Nosanchuk and Casadevall, 2003) have been associated with a reduction in their virulence.

In our study, *F*. *graminearum*, *C. graminicola*, *F. verticillioides*, *F. phaseoli,* and *M. phaseolina* have mycelial color turned white, suggesting a possible reduction in their virulence. Isaac (1994) postulated that fungi pigments might have originated from defense demands to fulfill a protective role in preventing the hydrolysis of their mycelium by enzymes produced by other microbes. Thus, fungal pigments confer tolerance to biotic and abiotic stresses and may result from synthesizing physiologically active byproducts (Avalos and Limón, 2015). Thus, besides the effect of secreted metabolites, it seems that VOCs of CNPMS-22, turning specific fungi to white, may reduce the virulence of at least five pathogenic fungi, adding another attribute to its role in plant protection.

Volatile organic compounds (VOCs) are byproducts of microbial metabolism that play essential roles in intra- and inter-kingdom interactions. These compounds are long-distance messengers produced by microorganisms’ primary and secondary metabolites (Schulz-Bohm et al., 2017). VOCs are evaporable and can readily travel through the air over long distances (El Jaddaoui et al., 2023). Various studies have identified several VOCs with potent antifungal activity in *B. velezensis*. For example, Li et al. (2020) found 30 volatile compounds in the strain CT32 with broad-spectrum antifungal activity. These authors reported that, upon exposure to VOCs emitted by the strain CT32, the mycelial growth of *Verticillium dahliae* and *Fusarium oxysporum* was reduced by 66.94 and 45.72%, respectively. In another study, Calvo et al. (2020) evaluated the *in vitro* and *in vivo* activity of the volatilome of three strains of biocontrol *B. velezensis* (BUZ-14, I3, and I5) against *Botrytis cinerea*, *Monilinia fructicola*, *M. laxa*, *Penicillium italicum*, *P. digitatum*, and *P. expansum*. These authors observed that the growth *in vitro* of these pathogens was significantly inhibited, in particular, *M. laxa*, *M. fructicola*, and *P. italicum* (66, 72 and 80%, respectively) by BUZ-14 and *B. cinerea* (100%) by I3 and I5. *In vivo* tests also showed significant inhibitions since volatile metabolites of I3 reduced gray mold in grapes by 50%, and those of BUZ-14 decreased brown rot severity in apricots, especially by *M. fructicola*, from 60 to 4 mm (Calvo et al., 2020). *Bacillus velezensis* CE 100 disrupted the cell membrane integrity of *Colletotrichum gloeosporioides* and reduced spore germination by 36.4% and mycelial growth by 20.0% (Choub et al., 2022). These authors also found that 5-nonylamine and 3-methylbutanoic acid suppressed the spore germination of *C*. *gloeosporioides* by 10.9 and 30.4% and reduced mycelial growth by 14.0 and 22.6%, respectively. Ling et al. (2022) identified 10 VOCs of *B. velezensis* L1 through headspace-gas chromatography-ion mobility spectrometry (GC-IMS) and pure chemical tests. They found that the *in vivo* and *in vitro* biocontrol effects of VOCs released by *B. velezensis* L1 on *Alternaria iridiaustralis* were 92.86 and 90.30%, respectively, and spore germination and sporulation were 66.89 and 87.96%, respectively. These authors also demonstrated that 2,3-butanedione had the most potent antifungal effects, inhibiting the growth of *A*. *iridiaustralis* in wolfberry fruit (Ling et al., 2022). Finally, Tang et al. (2024) found that the VOCs 2-dodecanone and 2-undecanone exhibited inhibition rates of 81.67 and 80.08%, respectively, against mycelial growth of *Sclerotium rolfsii* LC1. The present study evidenced the potent activity of VOCs against the 10 fungi tested. Volatile organic compounds of CNPMS-22 showed an inhibitory effect above 90% on the growth of *Aspergillus niger*, *A. ochraceus*, *Colletotricum graminicola*, and *Penicillium* sp. Volatile organic compounds also significantly reduced the mycelial densities of *Fusarium graminearum, Macrophomina phaseolina*, and *Stenocarpella maydis*. This series of studies revealed VOCs’ high diversity and complexity and their strain-specificity.

Volatile organic compounds produced by CNPMS-22 were more effective than DCP in controlling the growth of fungi, as evidenced by a comparison between DCP and ISP. For example, VOCs showed an inhibition rate above 90% for *Aspergillus niger*, *A. ochraceus*, *Colletotricum graminicola*, *Fusarium phaseoli*, and *Penicillium* sp. against 59.05, 60.71, 72.83, 75.14, and 71.64% in the DCP assay, respectively.

### 4.2 Scanning electron microscopy

Scanning electron microscopy (SEM) analysis of the mixed culture consisting of CNPMS-22 and *F*. *verticillioides* CML2778 revealed morphological damages in the fungus conidia, characterized by wrinkled hyphae with large depressions and loss of turgidity. The effective lysis of fungi’s cell walls is known to be associated with apoptosis of fungal cells induced by lipopeptide, secreted proteases, hydrolytic enzymes, and VOCS (Qi et al., 2010; Wu et al., 2023; Tian et al., 2022; Sun et al., 2024). Also, CNPMS-22 bacterium and biofilm adhered to hyphae of CML2778 were frequent. Thus, the morphological alterations in CML2778, evidenced by SEM, might be attributed to an additive effect of biofilm and a set of antifungal compounds produced by CNPMS-22.

### 4.3 Genome sequencing

The genome of CNPMS-22 showed high identity with the type strains of *B. velezensis* and *B. amyloliquefaciens*, confirming the high identity between both species. Together with *B. siamensis*, these species form the operational group of *B. amyloliquefaciens* of close-related species (Fan et al., 2017). In addition, these species belong to the *B. subtilis* complex, further complicating genomic analysis and making it challenging to curate databases.

The antifungal activity of *B. velezensis* has been frequently associated with three families of well-known cyclic lipopeptides (LPs), such as surfactin, fengycin, and iturin A (bacillomycin-D), encoded by three clusters of nonribosomal peptide (NRPs) genes (Koumoutsi et al., 2004; Meena et al., 2018; Yin et al., 2024). In a previous study, performing the UPLC-MS analysis, we found the presence of the three lipopeptides surfactin, fengycin, and iturin in culture supernatants of CNPMS-22 (Souza et al., 2018). In this study, we used the genomic data to perform manual searches and confirm the existence of the three clusters encoding surfactin, fengycin, and iturin in the CNPMS-22 genome. Genome analysis and mining also revealed that CNPMS-22 harbors other gene clusters encoding the biosynthesis of secondary metabolites of two main groups: non-ribosomal cyclic lipopeptides (NRPs) (bacillibactin and bacillomycin D) and polyketides (PK) (macrolactin, bacillaene difficidin, oxidifficidin, bacillaene, and macrolactin). The strain CNPMS-22 also shows ribosomally synthesized and post-translationally modified peptides (RiPPs) comprising bioactive peptides, enzymes, and volatile organic compounds (Supplementary Table S1).

We adopt a manual procedure to complement the computational analysis because the nomenclature of genes of *B. velezensis* using PROKKA sometimes generates conflicting results, making identifying targeted clusters difficult. For example, the search for iturin in many genomes of *B. velezensis* deposited in the GenBank fails because it is annotated simply as nonribosomal peptide synthetases, and one of them, among various, corresponds to iturin. In this scenario, the amino acid sequence of a target gene allows the correct identification of the gene of interest. Another complicating factor for using computational information to identify genes in a newly sequenced genome resides in synonyms for various genes and proteins, making accurate identification of genes difficult. In these cases, the amino acid sequence and protein domains are the best choice to determine the presence of a gene in the target genome, even with some differences.

The search for genes controlling the synthesis of volatile organic molecules and their precursors in the CNPMS-22 genome revealed 12 genes. However, most VOCs are byproducts of metabolic processes and can be determined only by gas chromatography–mass spectrometry (SPME-GC/MS) (Choub et al., 2022; Ling et al., 2022; Wu et al., 2023). In our study, VOCs of CNPMS-22 inhibited the mycelial growth of five fungi above 90%.

Various studies also reported the role of antibiotics, proteases and hydrolytic enzymes, biofilm, quorum sensing, and quorum quenching enzymes, among other factors, in the antifungal activity of *B. velezensis* (Xu et al., 2016; Borriss et al., 2019; Diniz et al., 2023; Liu et al., 2024). Also, a myriad of other compounds with antifungal activity have been identified in *B. velezensis*, like acetoin (3-hydroxybutanone) (Xiao and Lu, 2014; Zheng et al., 2023) and alcohols, benzenes, alkyls, ketones, aldehyde, alkene, ether and terpenes terpenes (Dhouib et al., 2019). These molecules and their derivatives have been extensively studied as potential alternatives to chemical fungicides in the cosmetics, food, and pharmaceutical industries (Ge et al., 2016; Kim et al., 2016; Han et al., 2021; Maina et al., 2022; Zheng et al., 2023). Modular polyketide synthase involved in the biosynthetic pathways of polycyclic aromatic products like macrolactins, bacillaenes, difficidin, bacilysin, and the siderophore bacillibactin were identified in various strains of *B. velezensis* (Koumoutsi et al., 2004; Das and Khosla, 2009; Rabbee and Baek, 2020; Wang et al., 2022; Liu et al., 2024; Rivers and Lowell, 2024).

Using manual analysis and bioinformatic, we found other genes with antifungal activity in the CNPMS-22 genome, such as extracellular lytic enzymes, chitinase, cellulase, *β*-1,3-1,4-glucanase, phosphodiesterases, extracellular proteases (dihydrolipoamide dehydrogenase, oxalate decarboxylase), subtilisin and serine proteases, as previously reported (Xu et al., 2016; Choub et al., 2021; Ning et al., 2021). These results reinforce the potential arsenal of CNPMS-22 and reveal that the antifungal activity of *B. velezensis* comprises various mechanisms controlled by many genes, forming a complex independent network with lipopeptides, secreted enzymes, VOCs, biofilm, siderophores, and ISR genes. However, the *in silico* genomic analysis, per se, showing the presence of several putative genes related to the antifungal activity observed in CNPMS-22 does not guarantee that they are active *in vivo*. Thus, expression analysis experiments using gene knockout, reverse transcription polymerase chain reaction (RT-PCR), quantitative polymerase chain reaction (qPCR), metabolomics, transcriptome, or proteomic are necessary for precisely identifying and quantifying the individual contribution of each of these genes to the antifungal activity of CNPMS-22. In addition, comparative studies of the CNPMS-22 genome with other well-characterized *B. velezensis*, such as the type strain FZB42 which differ of CNPMS-22 by 0.003%, may help elucidate the molecular aspects of CNPMS-22 phenotype.

### 4.4 Greenhouse and field experiments

The greenhouse experiment demonstrated the role of CNPMS-22 in protecting plants against *F*. *verticillioides* and other fungal diseases. In soil infested with the fungus, the detrimental effects of the fungus were evident, with plant survival of 34.37%. Conversely, seed treatments with fungicide or CNPMS-22 augmented the survival rates of plants to 87.5% and significantly improved the plants’ health. This result underscored the protective effects of CNPMS-22 and fungicide treatments on plants. The fungal disease symptoms in plants inoculated with CML2778 ranged from lesser to severe chlorosis, while CNPMS-22 significantly diminished the disease incidence, with an average reduction of 75% (*p* > 0.5).

In the field experiment, the plants from seeds treated with CNPMS-22 did not show symptoms of fusariosis disease. Concerning the field experiment, it is essential to highlight that it was done only in one season and locally since our primary objective was to certify that the antagonist activity of CNPMS-22, observed *in vitro*, could be replicated *in vivo* in the greenhouse and field conditions. This result established the foundation for future experiments to transform this strain into a bio-input for tropical maize crops. The Embrapa partnerships with private companies stated that, from that point, they assume the costs and design and implement new field experiments in the Brazilian maize-producing areas. Only after that can the bio input be commercialized.

In Brazil’s producing areas, the high incidence of diseases significantly reduces crop productivity. The increase in Brazilian productivity in recent years occurred due to the intensive use of agricultural inputs. In particular, the high indiscriminate use of fungicides has generated contamination problems for farmers and the environment. The incidence of stalk rot caused by *F*. *verticillioides* reaches over 70%, which causes productivity losses of up to 50% in susceptible cultivars under environmental conditions favorable to the pathogen’s development. This pathogen can cause disease symptoms at all stages of a plant’s development, causing seed losses, seedling death, stalk and ear rot, and damage to stored grains (Samsudin and Magan, 2016; Diniz et al., 2022b).

In developing countries, the high cost of chemical fungicides and their low effectiveness often reduce productivity and make agricultural activities more expensive and less profitable. In this study, CNPMS-22 inoculated in seeds, protected maize plants, equivalent to fungicide. Thus, the cost-effectiveness of CNPMS-22 may represent a promising solution for the high cost of fungicides, improving productivity and reducing expenses in these regions.

Fungal diseases often lead to reduced productivity and increased agricultural expenses. In these scenarios, the cost-effectiveness of CNPMS-22 and its high efficiency as an antifungal agent offer a promising contribution to minimizing the damages of fungal diseases and increasing productivity. In this complex scenario, a cost–benefit analysis must be carefully evaluated before introducing an inoculant with a foreign species into the microbiome.

In our study, the visual disease symptoms in plants grown in the greenhouse and field were an easy diagnostic tool for evaluating the CNPMS-22 antagonist activity against *F*. *verticillioides* since the contrast between plants from seeds inoculated with CNPMS-22 and CML2778 was evident. In the greenhouse experiment, the fungal disease symptoms were first observed 45–50 days after planting and ranged from a lesser to severe chlorosis. The experiments in the greenhouse and in field demonstrated the role of CNPMS-22 in protecting plants against *F*. *verticillioides* and other fungal diseases. In addition, in the field experiment, CNPMS-22 presented an activity similar to that of a chemical fungicide to protect plants against fungal diseases.

*Fusarium verticillioides* also produces fumonisins that can cause various health problems for humans and animals (Silva et al., 2009; Blacutt et al., 2018). *Fusarium verticillioides* can infect maize through the seeds, plant wounds, or stigma (Munkvold et al., 1997; Diniz et al., 2022a,b). The fungus impairs seed germination; root infection may cause plant death or drastically affect maize growth and yield (Munkvold et al., 1997; Diniz et al., 2022a). Alternative fungal control methods have been developed to minimize the risks of mycotoxins and residues of substances used to control fungi in grains and derivatives used in food.

Contamination of grains by fungi occurs mainly pre-harvest, causing ear rot (field fungi), or post-harvest (*Penicillium* and *Aspergillus*), generating moldy grains and mycotoxin accumulation during storage and processing under inadequate conditions (Lanza et al., 2014; García-Díaz et al., 2020; Yu and Pedroso, 2023). In Brazil, the principal genera of fungi in the field are *Fusarium*, *Cephalosporium*, *Gibberella*, *Nigrospora*, *Helminthosporium*, *Alternaria*, and *Cladosporium*. These fungi generally do not develop during storage, except in grains with high moisture content (Mannaa and Kim, 2017; Prestes et al., 2019).

Biological control of fungi using *Bacillus* sp. presents a series of advantages to chemical control, as it does not contaminate or cause environmental imbalance, does not leave residues in grains used as food, and is a cheap and easy-to-apply alternative (Jan et al., 2023; Lee et al., 2023; Karačić et al., 2024).

In addition, treating seeds with CNPMS-22 improved the germination and health of maize seedlings. Fungi species from *Fusarium*, *Aspergillus*, *Penicillium*, and Claviceps (not tested) produce toxins that can cause disease and death in humans and other animals. Mycotoxins can be present in stored grains and food as contaminants. Thus, seed inoculation with CNPMS-22 can protect stored grains, maintain their quality, and prevent grain contamination by mycotoxins.

The bacterial biofilm of plant growth-promoting rhizobacteria attached to the roots induces plant growth and exerts biocontrol activity, protecting plants from soil-borne pathogens (Bhattacharyya et al., 2023; Li et al., 2024; Rafique et al., 2024). Biofilm adhered to roots form a shield that excludes other organisms from the same niche and constitutes the initial stage of bacterial activity against pathogens (Haggag and Timmusk, 2008; Abd El Daim et al., 2015; Nucci et al., 2023). Thus, the plant-bacteria association through root colonization and biofilm formation is crucial for protecting plants from harmful fungi and competitors.

In nature, the involvement of biofilm in bacterial antagonism is strongly related to niche exclusion; biofilm-associated with plant roots is responsible for biocontrol since it occupies the pathogen colonization sites (Haggag and Timmusk, 2008; Schluter et al., 2015). The dense biofilm matrix limits the diffusion of compounds secreted by bacteria, which are therefore concentrated at the pathogen infection sites (Haggag and Timmusk, 2008; Abd El Daim et al., 2015). This environment is critical for accumulating antifungal lipopeptides and VOC compounds, improving the biocontrol activity of antagonistic bacteria. In our work, the strain CNPMS-22’s high ability to form biofilm and produce EPS was demonstrated *in vitro* by the Eppendorf assay, biofilm quantification in microtiter plates, EPS precipitation, and scanning electron microscopy. Bacteria attach to the root surface using various cell surface components, such as outer membrane proteins, wall polysaccharides (capsules), lipopolysaccharides, cell surface agglutinin, and exopolysaccharides. In addition, biofilm matrix components from *B. velezensis* are essential for trophic interactions in the microbial community and play a central role in driving its assembly and function (Sun et al., 2022). Our study identified various genes involved in plant colonization and biofilm formation in the CNPMS-22 genome.

CNPMS-22 suppresses fungal pathogens probably by secreting an array of lipopeptides, secondary metabolites, and hydrolytic enzymes in its surrounding environment. In addition, VOCs, biofilm formation, and the exclusion of competitors through colonization of plant roots must be involved in its antifungal activity. Finally, the induction of systemic resistance in plants (ISR) by VOCs, biofilm adhered to roots, colony forming, and regulation of the cellular transport of water and solutes contribute to plant growth and defense against pathogens (Tian et al., 2022; Kamalanathan et al., 2023; Sun et al., 2024). Thus, the ISR in plants is also an essential part of the biological control activity exerted by *B. velezensis*. Altogether, these complex bacterial networks allied to quorum sensing and quorum quench molecules protect plants from pathogenic microbes and modulate plant defense and growth.

The positive effect of CNPMS-22 on maize plants by inhibiting the development of fungal disease symptoms and promoting plant growth could be observed in the greenhouse and field experiments. In the greenhouse, CNPMS-22 inhibited growth and prevented the development of disease symptoms in soil infested with *F*. *verticillioides* while promoting an increase in plant height and dry matter. CNPMS-22 also prevented the development of fungal diseases in the field and increased the maize yield. These attributes of CNPMS-22 may contribute to improving crop production and indicate that this strain can become a player in integrated disease management strategies. However, several studies have warned about the risks of non-target effects of deliberately released organisms in a new environment due to their potential impact on native microbial communities and ecosystem functioning. Mawarda et al., 2020, state that the non-target effects of deliberately released organisms into a new environment are of great concern due to their potential impact on biodiversity, soil, and ecosystem functioning. However, *B. velezensis* is recurrently isolated from rhizosphere soil, phloem, leaves, and stigma from healthy maize and soybean plants cultivated in all Brazilian territories with diverse climates and ecosystems. In addition, various *Bacillus* species have been widely used in agriculture due to their diverse biological activities. Commercial formulations using *B. velezensis* are available on the biopesticide market (Fan et al., 2018; Shen et al., 2023; Zhong et al., 2024). The results demonstrating that *B. velezensis* CNPMS-22 has broad-spectrum antifungal and plant growth-promoting traits indicate that this bacterium could be a promising candidate for development into a commercialized product for sustainable agriculture.

## 5 Conclusion

The present study, *in vitro*, in the greenhouse and the field, showed relevant results concerning the antifungal activity of the strain CNPMS-22 and its plant growth promoter ability. The genome sequencing revealed that CNPMS-22 belongs to *B. velezensis*. CNPMS-22 displays three clusters of nonribosomal lipopeptides, genes for secondary metabolites, and hydrolytic enzymes with antifungal activity. The strain CNPMS-22 has excellent potential for developing inoculants to protect plants against pathogenic fungi and promote plant growth and yield. CNPMS-22 may suppress fungal pathogens by secreting an array of lipopeptides, secondary metabolites, and hydrolytic enzymes in its surrounding environment. CNPMS-22 may also reduce cost production by replacing total or partially the use of fungicides in agriculture and contribute to sustainable development and environmental preservation.

## Data Availability

The datasets presented in this study can be found in online repositories. The names of the repository/repositories and accession number(s) can be found at: https://www.ncbi.nlm.nih.gov/nuccore/?term=CNPMS-22JBHGBV000000000.1.
