# Supplementary material for: Bacillus velezensis CNPMS-22 as biocontrol agent of pathogenic fungi and plant growth promoter
Source: Front Microbiol. 2025 Mar 5;16:1522136. doi: 10.3389/fmicb.2025.1522136 (PMC11921153; doi:10.3389/fmicb.2025.1522136)
Supplement: Supplementary file 1 [file Data_Sheet_1.docx]

**Table S1. Genes involved in the synthesis of antifungal compounds and biofilm formation in the CNPMS-22 genome, and plant growth and colonization**

| non-ribosomal secondary metabolites (NRSM) | | | | | | |
| --- | --- | --- | --- | --- | --- | --- |
| Cluster | **Type** | **Gene and Synonyms** | **Cluster and genes** | **Function** | **Antimicrobial Activity** | **Reference** |
| Macrolactin H  polyketide | transAT-PKS | *Mnl* | mlnABCDEFGHI | Direct supression | Antibacterial  Antifungal | Nagao et al., 2001 |
| Difficidin  cyclic lipopeptide  polyketide | transAT-PKS  NRPS | *Dfn* | dfnAYXBCDEFGHIJKLM | Direct supression | Antibacterial  Antifungal | Wilson et al., 1987  Ye et al., 2018 |
| Bacillaene  polyketide | Polyketide + NRPS /  transAT-PKS, T3PKS, transAT-PKS-like, NRPS | *Bae* | baeBCDE, acpK, baeGHIJLMNRS | Direct supression | Antibacterial  Antifungal | Cheng et al., 2002 |
| Bacillibactin  2,3- dihydroxybenzoate (*dhb*)  lipopeptide | NRPS, RiPP-like | *Dhb* | dhbABCDEF | Siderophore production | Antibacterial  Antifungal  Iron-acquisition  Microbial competitors | May et al., 2001  Ye et al., 2018 |
| bacillomycin D from the Iturin group  lipopeptide | NRPS/PKS | *Bmy* | bmyCBAD | ISR | Antifungal | Landy et al., 1948  Delcambe and Devignat, 1957 |
| Surfactin  lipopeptide | NRPS | *Srf* | srfABCD | Biofilm  ISR | Biofilm  Antiviral  Antibacterial  Antifungal | Arima et al., 1968 |
| Bacilysin  dipeptide antibiotic | AMPs  NRPS | *Bac*  *Ywf* | bacABCDE, ywfG | Direct suppression | Antibacterial  Anti-Oomycetes  Antifungal | Foster and Woodruff, 1946 |
| Fengycin lipopeptide | NRPS, transAT-PKS, betalactone | *Fen* | fenABCDE | ISR | Antifungal | Vanittanakom et al., 1986 |
|  | | | | | | |
| ****Ribosomal synthesized antimicrobial compounds (RiPPs)**** | | | | | | |
|  |  |  |  |  |  |  |
| Plantazolicin  Cluster  peptide | ribosomally synthesized and post-translationally modified peptide (RiPP) | *Pzn* | pznFKGHIAJCDBEL | Direct suppression | Antibacterial  Antifungal  Nematicide | Scholz et al., 2011  Ye et al., 2018 |
| Amylocyclicin | ribosomally synthesized | *Can* | *acnBACDEF* | Direct suppression | Antifungal | Scholz et al., 2014  Cai et al., 2017 |
| nrs Cluster  lipopeptide | ribosomally synthesized Polyketides | *Nrs* | *nrsABCDEFG* | Putative peptide | Antibacterial | Chen et al., 2014 |
|  |  |  | Amylocyclicin/bacteriocin | uberolysin/carnocyclin family circular bacteriocin | Antimicrobial | Gabrielsen et al., 2014 |
|  |  | *lcI* | Lci - Antimicrobial peptide LCI,  SPBc2 prophage-derived uncharacterized protein yoqM | biocontrol | Antimicrobial | Vannier et al., 2019; 2023 |
| VOCs/ ISR |  |  |  |  |  |  |
|  |  | *acoA* | 2,6-dichlorophenolindophenol oxidoreductase subunit alpha | ISR | Antibacterial  Antifungal | Ryu et al., 2004  Raza et al., 2016  Rabbee et al., 2019 |
|  |  | *acoB* | acetoin: 2,6-dichlorophenolindophenol oxidoreductase subunit beta | ISR | Antibacterial  Antifungal | Ryu et al., 2004  Raza et al., 2016  Rabbee et al., 2019 |
|  |  | *acuC* | acetoin utilization protein AcuC | ISR | Antibacterial  Antifungal | Ryu et al., 2004  Raza et al., 2016  Rabbee et al., 2019 |
|  |  | *budA/*  *alsD* | acetolactate decarboxylase/ Alpha  acetolactate decarboxylase | ISR  Production of acetoin | Antibacterial  Antifungal | Ryu et al., 2004  Raza et al., 2016  Rabbee et al., 2019 |
|  |  | *alsS* | acetolactate synthase AlsS  pH6 acetolactate synthase | ISR  Production of acetoin | Antibacterial  Antifungal | Ryu et al., 2004  Raza et al., 2016  Rabbee et al., 2019 |
|  |  | *ilvN* | acetolactate synthase small subunit | ISR  Production of acetoin | Antibacterial  Antifungal | Ryu et al., 2004  Raza et al., 2016  Rabbee et al., 2019 |
|  |  | *ilvB* | acetolactate synthase large subunit | ISR  Production of acetoin | Antibacterial  Antifungal | Ryu et al., 2004  Raza et al., 2016  Rabbee et al., 2019 |
|  |  | *alsR* | als operom regulatory protein  LysR family transcriptional regulator/  HTH-type transcriptional regulator AlsR | ISR  Production of acetoin | Antibacterial  Antifungal | Ryu et al., 2004  Raza et al., 2016  Rabbee et al., 2019 |
|  |  | *alsT* | Amino-acid carrier protein alsT,  Na+/alanine symporter | ISR  Production of acetoin | Antibacterial  Antifungal | Ryu et al., 2004  Raza et al., 2016  Rabbee et al., 2019 |
|  |  | *bdhA* | 2,3-Butanediol dehydrogenase | ISR  Production of acetoin | Antibacterial  Antifungal | Ryu et al., 2004  Raza et al., 2016  Rabbee et al., 2019 |
| Biofilm formation |  |  |  |  |  |  |
|  |  | *iolW*  *iolG*  *iolU*  *yulF* | inositol 2-dehydrogenase  Scyllo-inositol 2-dehydrogenase (NADP^+^)  intermediary metabolismo  protein Gfo/Idh/MocA family oxidoreductase | Metabolism of carbohydrates and related molecules  Myo-inositol catabolism | involved in biofilm formation protein | Yoshida and Kuramitsu, 2002 |
|  |  | ywcE | spore morphogenesis  germination protein YwcE  cytochrome aa3 quinol oxidase subunit IV | GNAT family N-acetyltransferase |  |  |
|  |  | *bslA*  *yuaB* | Glu/Leu/Phe/Val dehydrogenase | Biofilm surface layer hydrophobin BslA |  | Kobayashi and Iwano, 2012  Hobley et al., 2013  Xie et al., 2024 |
|  |  | *bslB*  *spsL* | biofilm surface layer hydrophobin BslB  dTDP-4-dehydrorhamnose 3,5-epimerase Family protein and relate enzymes | Biofilm hydrophobicity  Biofilm surface layers and assembly |  | Hobley et al., 2013 |
|  |  | *wecB*  *mnaR,A* | UDP-Glc - UDP-N-acetylglucosamine 2-epimerase (non-hydrolyzing) | Lipopolysaccharide biosynthesis  Precursor to WTA polymers  Colony morphology  Exopolysaccharides  Cell wall/membrane/envelope biogenesis |  | Xu et al., 2019  Martín-Rodríguez et al., 2021 |
|  |  | *piA* | Acyltransferase family protein (three genes) | Involved in polysaccharide intercellular adhesin (PIA) |  | Zhu et al., 2007 |
|  |  | *Spo0A* | Sporulation transcription factor Spo0A  the master transition state regulator  Stage 0 sporulation protein A  Response regulators consisting of a CheY-like receiver domain and a winged-helix DNA-binding domain as well as for solid surface-associated biofilm formation | Spo0A Expression is associated with abrB  DegU and Spo0A jointly control transcription of two loci required for complex colony development by *Bacillus subtilis*  regulator activity - regulation of DNA-templated transcription regulation of sporulation resulting in formation of a cellular spore  key for biofilm formation  Spo0A is required positively for pellicle formation and the development of complex architecture |  | Branda et al., 2001  Hamon and Lazazzera, 2001  Meliawati et al., 2022 |
|  |  | *sinI*  (two different genes) | anti-repressor SinI family protein | the Master Regulator of Biofilm Formation in *Bacillus subtilis*, inhibitor of *sinR*  antagonist of SinR - sinI - biofilm formation, development, and regulation - Acts as an antagonist to SinR. SinI prevents SinR from binding to its target sequence on the gene for AprE. Contains 1 Sin domain |  | Newman et al., 2013 |
|  |  | *sinR* | XRE family transcriptional regulator  sinR - transcriptional regulator for post-exponential-phase response  tapA-sipW-tasA operon | master regulator for biofilm formation  biofilm formation, development, and regulation  formes wrinkled microcolony  Acts as an antagonist to SinI |  | Dergham et al., 2021 |
|  |  | *tasA* | TasA family protein  tapA-sipW-tasA operon | major protein component of the biofilm extracellular matrix  functional amyloid protein that forms resistant fibers that confer structural stability to biofilms.  matrix production |  | Branda et al., 2001  Xie et al., 2024 |
|  |  | *yqxM* | YqxM | lipoprotein for biofilm formation |  | Meliawati et al., 2022 |
| root colonization determinants |  |  |  |  |  |  |
|  |  | *typA* | translational GTPase TypA | root colonization determinants,  GtP binding protein, root colonization,  Response to stress,  Predicted membrane GTPase involved in stress response |  | Vannier et al., 2023 |
| Operon *pstBACS*  *pstBBACS* in *B*. *velezensis* |  | *pstA* | phosphate ABC transporter permease PstA | ATP-binding cassette (ABC) transporter complex,  substrate-binding subunit, plasma membrane, root colonization  ATPase-coupled phosphate ion transmembrane transporter activity, root colonization |  | Vannier et al., 2023 |
|  |  | *pstB*  two genes | phosphate ABC transporter ATP-binding protein PstB | ATP-binding cassette (ABC) transporter complex,  substrate-binding subunit, plasma membrane  ATPase-coupled phosphate ion transmembrane transporter activity, cytoplasmic side of plasma membrane  root colonization |  | Vannier et al., 2023 |
|  |  | *pstC* | phosphate ABC transporter permease subunit PstC | ABC-type phosphate transport system  Permease component  Root colonization |  | Vannier et al., 2023 |
|  |  | *pstS* | Phosphate-binding protein pstS 1 PBP 1 | ABC-type phosphate transport system  Periplasmic component  Root colonization |  | Vannier et al., 2023 |
|  |  | *yobO1* | Pre-neck appendage protein | Autotransporter adhesin  Root colonization | involved in the degradation of the EPS biofilm matrix | Borriss et al., 2011 |
|  |  | *yobO2* | Pre-neck appendage protein,  peptidase G2 autoproteolytic cleavage domain-containing protein | Autotransporter adhesin  Root colonization | autotransporter adhesin | Hao et al., 2012 |
|  |  | *mtnA* | Manganese ABC transporter substrate-binding lipoprotein MtnA,  zinc ABC transporter substrate-binding protein  S-methyl-5-thioribose-1-phosphate isomerase | ABC-type metal ion transport system  Periplasmic component/surface adhesin/ root colonization | Periplasmic component/surface adhesin" | Meléndez et al., 2020 |
|  |  | *Ycdh* | putative zinc transport system zinc-binding lipoprotein adcA | ABC-type metal ion transport system  Periplasmic component/surface adhesin / root colonization | homologue of adhesion protein precursor of *Streptococcus pneumonia* | Luo et al., 2021 |
|  |  | *ydhD*  *chiB* | chitinase |  |  | Tran et al., 2022 |
|  |  |  | S8 family peptidase | S8 family peptidase = Serine protease = intracellular serine  protease = Alkaline Serine Protease = chitinase, nematicide |  | Yang et al., 2020 |
|  |  | *YaaH*  *chiA* | glycoside hydrolase = glycosyl hydrolase (16 genes)  glycoside hydrolase family 18 protein = spore gernimation  protein YaaH - LysM peptidoglycan-binding domain-containing  protein | Chitinase | Antifungi, Nematicide | Tran et al., 2022 |
|  |  | *idA / iucD / pvdA* | IdA/IucD/PvdA family monooxygenase | Rhizobactin siderophore biosynthesis protein | siderophore | Kügler et al., 2020 |
|  |  | Three genes | Cellulase M - M42 family peptidase | Root colonization |  | Sharma et al., 2019 |
|  |  | *yoaC* | Sugar kinase, FGGY-family carbohydrate kinase,  cellulase Family, glycosylhydrolase, endo-beta-1,4-glucanase,  sugar kinase, 2-keto-3-deoxygluconokinase |  |  | Fan et al., 2016;  Zhang et al., 2011 |
|  |  |  | Glycoside hydrolase family 5 = anteriormente chamada de Cellulase family A  Cellulase - glycosyl hydrolase family 5 - Carbohydrate metabolism;  Cellulose degradation; Glycosidase; Hydrolase; Polysaccharide  degradation; Signal; belongs to the glycosyl hydrolase 5 (cellulase A)  family; contains 1 CBM3 (carbohydrate binding type-3)domain;  endoglucanase - BglC - extracellular enzyme -  cellulase family glycosylhydrolase |  |  | Fan et al., 2016 |
|  |  | *Bglu* | glycoside hydrolase family 16 protein, endo-beta-1,3-1,4  glucanase; Beta-glucanase/Beta-glucan synthetase | degradation of polysaccharides |  | Viborg et al., 2019 |
|  |  | *BglC* | Glycoside hydrolase family 5 = anteriormente chamada de Cellulase family A  Cellulase - glycosyl hydrolase family 5 - Carbohydrate metabolism;  Cellulose degradation  Glycosidase; Hydrolase; Polysaccharide degradation; Signal belongs to the glycosyl hydrolase 5 (cellulase A)  family; contains 1 CBM3 (carbohydrate binding type-3) domain  endoglucanase  BglC extracellular enzyme  cellulase family glycosylhydrolase | degradation of polysaccharides |  | Aspeborg et al., 2012 |
| Plant growth promoter |  |  |  |  |  |  |
|  |  | *pabB* | *IAA* - Indole-3-Acetic Acid Biosynthesis  anthranilate/para-aminobenzoate synthases component I  aminodeoxychorismate synthase, subunit I |  |  | Fässler et al., 2010 |
|  |  | *pabA* | Indole-3-Acetic Acid Biosynthesis  aminodeoxychorismate/anthranilate synthase component II |  |  | Fässler et al., 2010 |
|  |  | *menF* | - Isochorismate synthase EntC [Coenzyme transport and  metabolism, Secondary metabolites biosynthesis, transport and  catabolism - menaquinone-specific isochorismate synthase, | siderophore, plant growth promoter |  |  |
|  |  | *yucG* / *lpmo10* | Chitin binding protein AA10 protein = chitin-binding protein  Family 18 chitinase | yucG  Uncharacterized protein conserved in bacteria  lytic polysaccharide monooxygenase - chitin binding protein-  enhances the activity of insecticidal proteins and is fungistatic - facilitate propagation of *Bacillus* strain in environment by inhibiting growth of certain fungi |  | Manjeet et al., 2013 |
|  |  | *yvgO* | stress protein YvgO | Sodium, potassium, lithium and rubidium/H(+) antiporter |  |  |
|  |  | *cshA* | ATP-dependent RNA helicase CshA | Saline estresse |  | Kim et al., 2016 |
|  |  | *phoP* | Alkaline phosphatase | Phosphate metabolism |  | Zhang et al., 2023 |
|  |  | *phoD* | alkaline phosphatase D/ Phosphodiesterase | phosphodiesterase/alkaline phosphatase, degrades wall teichoic  acid during phosphate starvation |  | Zhang et al., 2023 |
|  |  | *FeuB* | ABC-type Fe3+-siderophore transport system | siderophore transport |  | Delepelaire, 2019 |
