# Supplementary material for: Bacillus velezensis CNPMS-22 as biocontrol agent of pathogenic fungi and plant growth promoter
Source: Front Microbiol. 2025 Mar 5;16:1522136. doi: 10.3389/fmicb.2025.1522136 (PMC11921153; doi:10.3389/fmicb.2025.1522136)

**MATERIAL AND METHODS**

1**. ASSESSMENT OF NITROGEN FIXATION POTENTIAL**

The bacterial strain was evaluated for atmospheric nitrogen fixation capacity in a triplicate nitrogen-free semisolid culture medium (Baldane et al., 1986). Tubes containing 3 mL^-1^ of semisolid NFb medium were inoculated in triplicate with 10 µL of bacterial culture at a contraction of 10^8^ UFC mL^-1^ (OD540nm= 1.0). After 5 days of incubation at 30°C, the strain that showed a visible film of growth below the surface of the medium with a change in color from green to blue was considered nitrogen-fixing.

**2. QUALITATIVE ASSESSMENT OF SIDEROPHORE PRODUCTION**

Bacterial strains were inoculated in triplicate in Petri dishes containing nutrient agar medium and incubated at 30°C for 16 h to evaluate siderophore production. Then, each plate received a thin layer of Overlay-CAS medium (Schwyn & Neilands, 1987), followed by incubation at 25°C for 4 days. Siderophore producers were considered microorganisms capable of promoting color changes in the culture medium.

**3. GROWTH OF CNPMS-22 IN MEDIA WITH REDUCED WATER ACTIVITY**

The Bacillus strain was inoculated into 10% (w/v) Tryptone Soy Agar (TSA) culture medium supplemented with 405 g/L sorbitol at 30 °C for 72 h, corresponding to a water activity value (Aw) of 0.919, in order to evaluate the growth of strains in medium with reduced water activity.

**4. SALINITY TOLERANCE (NaCl)**

The strain CNPMS-22 was inoculated in LB medium plus 10% and 20% (w/v) NaCl, and cultivated for 48 h at 30 °C for visual assessment of the bacterial growth to evaluate tolerance levels to sodium chloride (NaCl).

**5. QUANTITATIVE ASSESSMENT OF INDOL-ACETIC ACID (IAA) PRODUCTION**

IAA production was determined using the colorimetric method described by Patten and Glick (1996), with modifications. 1 mL of TSB medium supplemented with DL-tryptophan at a concentration of 1.0 g L^-1^ was added to the sterile Deep Well plate, followed by inoculation with 5 µL of bacterial culture (108 CFU.mL^-1^; OD540nm= 1.0). The plate was incubated at 30 °C in the dark at 100 rpm for 120 hours. After the time had elapsed, the plate was centrifuged at 4000 rpm for 15 minutes, and 100 µL of the supernatant was mixed with 100 µL of Salkowski reagent (49 mL of 35% (v/v) perchloric acid and 1 mL of 0.5 M FeCl_3_), followed by incubation for 20 minutes in the dark. The IAA concentration was estimated by reading the OD at 540 nm on a UV/VIS spectrophotometer (FLUOstar Omega, BMG LABTECH, Germany). The experiment was carried out in triplicate, and the formation of a pink color was shown to have a positive result. The concentration of IAA in the culture medium was determined by comparison with the standard curve made from commercial IAA at concentrations 0, 10, 20, 40, 80, and 100 µg mL^-1^_._

**6. PHOSPHATE SOLUBILIZATION AND MINERALIZATION**

Aliquots of 100 μL of the strain culture standardized at 10^8^ CFU/mL were inoculated in triplicate into NIBRIP solid culture media [10 g glucose, 5 g Ca_3_(PO4)_2_, 5 g MgCl_2_.6H_2_O, 0.25 g of MgSO_4_.7H_2_O, 0.2 g of KCl and 0.1 g of (NH_4_)2SO_4_] (Nautiyal, 1999) and in Phytate medium (Richardson et al., 2001), containing a source of P in the form of tricalcium phosphate and sodium phytate, respectively. The inoculated media were incubated at 28 °C for 10 days to visualize the transparent halo around the colonies, indicating P solubilization/mineralization. The experiment was carried out in triplicate, and the negative control consisted of using a culture medium without an inoculum. The diameter of the solubilization halo, perceived as a translucent area around the colony, was measured using a digital caliper. The Solubilization Index (SI) expressed the result using the formula: SI = ϕ Halo (mm)/ϕ Colony (mm) (Berraquero et al., 1976).

**7. Results**

**In vitro characterization of the *Bacillus velezensis* strain (CPMS-22) regards the main mechanisms associated with plant growth promotion.**

| ***In vitro* tests** | **CNPMS-22** |
| --- | --- |
| Nitrogen fixation | + |
| Biofilm Production**^1^** | +++ |
| Siderophore production**^2^** | + |
| Exopolysacharide (EPS) production | + |
| Hydric stress | +++ |
| Salinity resistance | + |
| IAA production (µg mL^-1^) | 42.76 |
| Solubilization index (SI), inorganic phosphorus solubilization (phytate)**^3^** | 1.81 |
| Inorganic phosphorus solubilization (NBRIP)^3^ | 1.44 |
| Phosphate tricalcium solubilization (mg L^-1^) | 73.27 |

The positive (**+**) and negative (**-**) signs signify the result for each growth-promoting characteristic evaluated.

**^1^ +++** Strongly activity or biofilm-forming, **++** Moderately activity or biofilm-forming, **+** Weakly activity or biofilm forming.

**^2^** Carboxylate-type siderophore.

**^3^**P solubilization and mineralization index in Phytate and NBRIP culture medium, containing organic and inorganic phosphorus, respectively.

**8. Phosphate solubilization**


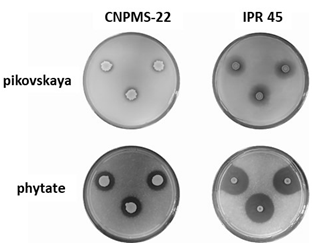


**Figure S1. Phosphate solubilization by CNPMS-22. IPR 45, strain of *Pseudomonas aeruginosa*.**

**9. Nitrogen Fixation**

**
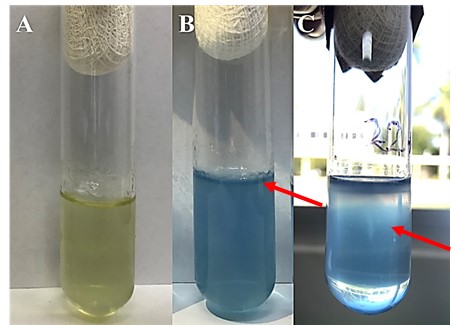
**

**Figure S2. A-** Control, **B** and **C,** CNPMS-22 growing in N-free semisolid medium. The arrows show a thin film and changes in the medium color, indicating nitrogen fixation by CNPMS-22.

**10. Plant growth promoter effect of CNPMS-22.**

**
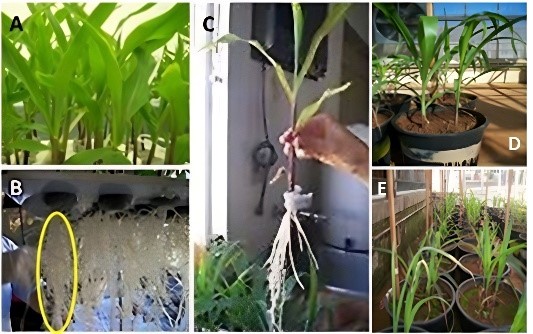
**

**Figure S3. Maize plants from seeds inoculated with CNPMS-22 growing in a growth chamber and greenhouse.** Plants in V4-V5 stages (25-30 days). **A** and **B**, plants from seeds inoculated with CNPMS-22 growing in a growth chamber, **C**, plant control without inoculation, **D,** plants inoculated, and **E**, plant not-inoculated.

**References**

Berraquero, F.R., Baya, A.M., Cormenzana, A.R. (1976) Establecimiento de índices para el estudio de la solubilización de fosfatos por bacterias del suelo. Ars Pharmaceutica. 17(4): 399-406. <https://revistaseug.ugr.es/index.php/ars/article/view/24869>.

Baldani, J.I., Baldani, V.L.D., Seldin, L., Döbereiner, J. 1986 Characterization of *Herbaspirillum seropedicae* gen. nov., sp. nov., a root-associated nitrogen-fixing bacterium. Int J Syst Bacteriol. 36: 86-93. <https://doi.org/10.1099/00207713-36-1-86>.

Patten, C.L, Glick, B.R. (1996) Bacterial biosynthesis of indole-3-acetic acid. Can. J. Microbiol. 42: 207-220. <https://doi.org/10.1139/m96-032>.

Schwyn, B., Neilands, J.B. (1987) Universal chemical assay for the detection and determination of siderophores. Anal Biochem. 160(1): 47-56. <https://10.1016/0003-2697(87)90612-9>.

**Figure S4. Climate data during the field experiment.**


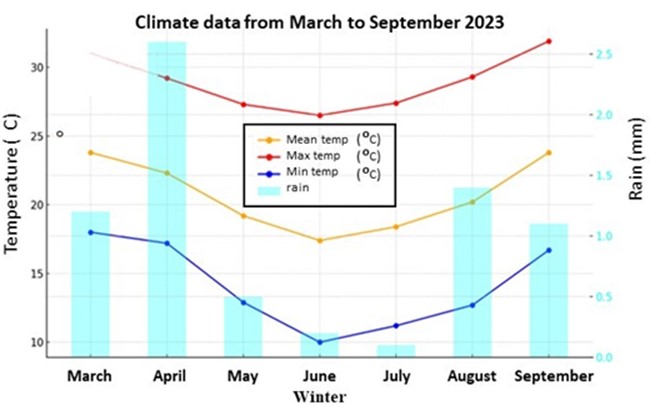

Supplement: Supplementary file 2 [file Data_Sheet_2.docx]
